# Implementation of an Electronic Medical Record-Embedded Refeeding Risk Order Set and Its Impact on Refeeding Syndrome Among Adults Receiving Enteral Nutrition: A Retrospective Cohort Study in an Inpatient Hospital Setting

**DOI:** 10.3390/nu18020226

**Published:** 2026-01-11

**Authors:** Emma Peterson, Audrey Arnold, Kristen Payzant, Leslie Wills, Mariah Jackson, Corri Hanson, Megan Timmerman, Rachel Lietka, Kaiti George, Jana Ponce

**Affiliations:** 1 College of Allied Health Professions, University of Nebraska Medical Center, Omaha, NE 68198, USA; epeterson01@unmc.edu (E.P.); auarnold@unmc.edu (A.A.); mariah.jackson@unmc.edu (M.J.); ckhanson@unmc.edu (C.H.); megan.timmerman@unmc.edu (M.T.); rlietka@unmc.edu (R.L.); 2 Department of Clinical Nutrition, Nebraska Medicine, Omaha, NE 68198, USA; kpayzant@nebraskamed.com (K.P.); lesevans@nebraskamed.com (L.W.); 3 Department of Kinesiology and Sport Science, University of Nebraska at Kearney, Kearney, NE 68849, USA; georgekd@unk.edu

**Keywords:** refeeding syndrome, refeeding risk, electronic medical record (EMR), order set, enteral nutrition (EN), malnutrition, electrolyte abnormalities, quality improvement

## Abstract

**Background/Objectives:** Refeeding syndrome (RFS) is challenging to prevent and manage in hospitalized patients receiving enteral nutrition (EN). Nebraska Medicine implemented an Electronic Medical Record (EMR) Refeeding Risk Order Set (RROS) to standardize prevention measures, including structured electrolyte monitoring, thiamine supplementation, and conservative EN initiation. This study evaluated whether RROS implementation reduced RFS occurrence or severity and assessed its operational impact. **Methods:** In this retrospective cohort study, adults receiving EN before and after RROS implementation were compared. Primary outcomes were RFS occurrence and severity; secondary outcomes included EN initiation and advancement rates, electrolyte trends, lab frequency, and electrolyte repletion. **Results:** RFS occurrence did not differ significantly between groups (92.3% vs. 91.3%, *p* = 0.694), nor did severity (*p* = 0.535). The post-RROS group received more electrolyte boluses on EN Day 0 (*p* = 0.027) and had a lower EN starting rate (15.7 vs. 18.3 mL/h, *p* = 0.045). **Conclusions:** Although the RROS did not reduce RFS occurrence or severity, integrating American Society for Parenteral and Enteral Nutrition (ASPEN)-based guidance into the EMR was highly feasible and adopted immediately. Automating electrolyte monitoring, micronutrient supplementation, and conservative feeding initiation reduces the risk of errors and promotes consistent care. These benefits improve workflow efficiency and support providers during high census periods, limited staffing, or when experience varies. Future research should explore combining EMR tools with predictive analytics to optimize early risk identification and individualized management.

## 1. Introduction

Refeeding syndrome (RFS) is a potentially life-threatening metabolic complication that develops when glucose is reintroduced after periods of inadequate intake, leading to insulin-driven intracellular shifts of phosphorus (Phos), potassium (K), and magnesium (Mg), as well as thiamine depletion [1]. These disturbances can cause cardiac, respiratory, and neurologic dysfunction and increase the risk for adverse outcomes, particularly among hospitalized patients with malnutrition. Despite its clinical relevance, RFS remains difficult to identify due to nonspecific symptoms and historically inconsistent diagnostic criteria [2]. Reported incidence varies widely from 0% to 62%, reflecting differences in definitions, monitoring practices, and management strategies [3].

In 2020, the American Society for Parenteral and Enteral Nutrition (ASPEN) published consensus criteria defining RFS based on decreases in electrolyte levels up to five days following nutrition support initiation [1]. In addition, observational studies continue to show that RFS is associated with increased mortality in malnourished medical inpatients, higher morbidity in older adults, and electrolyte instability among neurocritically ill patients [4,5]. Even with clearer diagnostic parameters, adherence to recommended prevention strategies like conservative nutrition support initiation, early electrolyte monitoring, and thiamine supplementation, remains inconsistent in routine practice, putting patients at higher risk for adverse outcomes [6].

Although several studies have assessed management of RFS and enteral nutrition (EN) initiation strategies, none have examined the real-world impact of embedding a standardized RFS prevention or management protocol directly into the electronic medical record (EMR). To address practice variability and enhance patient safety, Nebraska Medicine, a large, Midwest academic medical center located in Omaha, Nebraska, successfully implemented an EMR-embedded Refeeding Risk Order Set (RROS) in 2023. To support both registered dietitians (RDs) and non-dietitian practitioners, the RROS integrates key protocol elements, including conservative EN initiation at 10 mL/h, structured EN rate advancement, 8-h electrolyte monitoring, and thiamine supplementation.

The purpose of this study is to evaluate whether implementation of the EMR-embedded RROS reduced the incidence and severity of RFS among hospitalized adults receiving EN, using ASPEN criteria to standardize outcome assessment. We hypothesize that the implementation of the RROS will show a decrease in RFS occurrence and reduction in severity of RFS in the inpatient population.

## 2. Materials and Methods

### 2.1. Study Design and Setting

This retrospective cohort study evaluated the impact of an EMR-embedded RROS on the occurrence and severity of RFS among adult inpatients receiving enteral nutrition EN at a large Midwest academic medical center. The RROS incorporated an institutional protocol for identifying and managing patients at risk for RFS into EN ordering panels, based on ASPEN’s consensus statement [1]. The order set streamlined electrolyte replacement, EN advancement, biochemical monitoring, and micronutrient supplementation. Data were extracted from the EMR using a standardized report developed with a senior EMR analyst, utilizing a de-identified dataset as part of a quality improvement initiative.

Because the RROS did not exist prior to implementation, a true control group was not feasible. To approximate a high-risk comparison group, the pre-RROS cohort was limited to patients with a documented diagnosis of malnutrition who received EN between 2 May 2022, and 8 May 2023. This approach was chosen because malnutrition is a significant risk factor for RFS. In contrast, the post-RROS group included all patients for whom the RROS was selected and ordered between 9 May 2023, and 8 May 2024, regardless of malnutrition status, reflecting the real-world intent of the order set to support any patient at refeeding risk.

### 2.2. Intervention: EMR Refeeding Risk Order Set

When ordering EN, providers and registered dietitian nutritionists (RDNs) select either “standard continuous feeds” or “refeeding risk orders” (RROS). Providers were encouraged to use the RROS for patients diagnosed with or suspected of having malnutrition. Selecting the RROS automatically adds: the chosen EN formula, 100 mg oral thiamine daily, 1–18 mg multivitamin oral tablet with iron and folic acid, a basic metabolic panel (BMP) twice daily, magnesium labs twice daily, and inorganic phosphorus labs twice daily. Providers may remove labs or supplements based on clinical judgment.

Although not embedded in the RROS, the protocol recommends checking serum phosphorus before EN initiation and replacing levels < 2.0 mg/dL prior to feeding. EN typically starts at 10 mL/h and advances by 10 mL every eight hours to the patient-specific goal rate if electrolytes remain stable. This schedule is noted for refeeding risk but not auto-selected. EN is held or reduced by 50% if severe electrolyte disturbances occur and resumed when levels normalize.

### 2.3. Study Population

Participants were drawn from the RROS dataset. The pre-RROS group consisted of adult inpatients with a documented malnutrition diagnosis who received EN prior to RROS implementation, while the post-RROS group included all adult inpatients for whom the RROS was applied at EN initiation. This design reflects the operational use of the RROS.

Eligibility criteria: age ≥ 19 years, EN during hospitalization without parenteral nutrition, and documented serum magnesium, phosphorus, and potassium at admission, day of EN initiation (Day 0), and within five days after initiation. Patients were excluded if younger than 19 years or missing required measurements.

### 2.4. Data Collection

Data were obtained from a standardized electronic report generated by the health system’s IT department for this quality improvement project. Extracted variables included demographics (age, sex, height, weight, BMI, malnutrition diagnosis, MST score, LOS, ICU admission), nutrition parameters (EN formula type and daily infusion rates for days 0–5), and biochemical indices (serum Mg, Phos, K at admission and EN days 0–5). The total number of electrolyte boluses administered during the first five days of EN was recorded. An “electrolyte bolus” was defined as intravenous administration of potassium chloride, magnesium sulfate in dextrose, potassium phosphate, or sodium phosphate, regardless of dose.

### 2.5. Outcomes

The primary endpoint was the occurrence of RFS, defined according to the ASPEN consensus criteria as at least a 10% reduction in one or more of serum Phos, K, or Mg levels within five days of initiating EN [1].

Secondary outcomes included the severity of RFS, categorized as mild (10–20% reductions), moderate (20–30% reduction), or severe (greater than 30% reduction) based upon ASPEN criteria), the total number of electrolyte boluses administered during the first five days of feeding, and the mean starting EN infusion rate and mean EN advancement rate during the same period [1]. Secondary outcomes also included comparison of adherence to the RFS protocol with and without use of the RROS. Those outcomes included EN initiation rate and advancement rates, number of electrolyte lab draws, change in electrolyte levels, and number of electrolyte boluses.

### 2.6. Statistical Analysis

All statistical analyses were conducted using IBM SPSS Statistics (version 29.0.2). Baseline characteristics were analyzed using independent samples *t*-tests for continuous variables and chi-squared tests for categorical variables. The primary outcome, RFS occurrence, was compared between groups using a chi-squared test. Differences in RFS severity were evaluated using one-way analysis of variance (ANOVA). Associations between order set implementation and both the average change in daily electrolyte values and mean tube feeding rate were examined using *t*-tests. Associations between order set implementation and both electrolyte bolus administration and the number of daily lab draws were analyzed using chi-squared tests, with Fisher’s exact test applied when expected cell counts were five or fewer. Statistical significance was defined as a two-tailed *p*-value < 0.05.

## 3. Results

A total of 482 patients met inclusion criteria and were included in the final analysis. One patient was excluded due to age < 19 years old, and 824 were excluded for missing required electrolyte values. The overall cohort had a mean age of 60.7 years, an average BMI of 26.7 kg/m^2^, and a median LOS of 23.1 days. Approximately 74.5% were diagnosed with malnutrition, and 79.3% required ICU admission (Table 1). This cohort represents a highly complex inpatient population, with the majority requiring ICU-level care and diagnosed with malnutrition.

Of the final cohort, 207 patients were in the pre-RROS group and 275 in the post-RROS group. The groups did not differ significantly in age, MST score, or LOS; the mean age was 61.8 versus 59.8 years and LOS was 22.3 versus 24.0 days in the pre- and post-RROS groups, respectively (Table 1). However, the post-RROS group had a higher average BMI (*p* = 0.02), a lower prevalence of malnutrition (*p* < 0.001), and a higher ICU admission rate (*p* = 0.02) (Table 1). Differences in malnutrition prevalence reflect the study design: the pre-RROS group required a malnutrition diagnosis, while the post-RROS group included all patients for whom the RROS was applied, regardless of malnutrition diagnosis.

### 3.1. RFS Occurrence and Severity

There was no significant difference in RFS occurrence or severity between groups. RFS occurred in 191 (92.3%) of patients in the pre-RROS group and 251 (91.3%) in the post-RROS group (*p* > 0.05) (Table 2).

No significant differences in average change in electrolyte levels (K, Mg, and Phos) each day following EN initiation (Table 3). Of note, Phos changes on day 0–1 were decreased between pre-RROS −0.311 and post-RROS −0.157 (*p* = 0.146). Day 1–2 changes in Phos were increased between pre-RROS −0.137 and post-RROS −0.268 (*p* = 0.125). Day 2–3 changes in Phos were also increased between pre-RROS group −0.046 and post-RROS group −0.116 (*p* = 0.060). Day 3–4 changes in Mg were also increased between pre-RROS group 0.030 and post-RROS group −0.018 (*p* = 0.079). Day 4–5 changes in K were also increased between pre-RROS group 0.062 and post-RROS group −0.011 (*p* = 0.093) (Table 3).

### 3.2. Laboratory Monitoring and Electrolyte Replacement Patterns

When comparing the number of lab draws for K, Mg, and Phos each day between groups, on Day 2, the pre-RROS group had a higher percentage of K lab draws with 202 (99.0%) compared to the post-RROS group with 256 (93.09%, *p* = 0.33) (Table 4). Mg lab draws were also higher on Day 2 in the pre-RROS group with 197 (95.2%) compared to the post-RROS group with 245 (89.09%, *p* = 0.017) (Table 4). It is also of note that for both groups, on EN Day 0 there were 100% lab draws, meaning each patient had at least 1 lab draw for K, Mg, and Phos (Table 4).

Analysis of the number of electrolyte boluses administered each day showed that the post-RROS group had a higher number of boluses on EN Day 0, with 79 single electrolyte boluses, 13 double electrolyte boluses, 7 triple electrolyte boluses, and 0 quadruple electrolyte boluses, when compared to the pre-RROS group that had 52 single, 9 double, 0 triple, and 3 quadruple electrolyte boluses, respectively (*p* = 0.03) (Table 5). All other days did not show significant differences in frequency of electrolyte bolus administration.

### 3.3. EN Initiation and Advancement

The average EN start rate was significantly lower in the post-RROS group with a mean rate of 15.7 ± 7.9 mL/h compared to the pre-RROS group with a mean rate of 18.3 ± 14.0 mL/h, *p* = 0.045 (Table 6). There were no significant differences in EN rate advancement during the first five days of EN.

## 4. Discussion

This retrospective cohort study evaluated the implementation of an EMR-embedded Refeeding Risk Order Set (RROS) designed to standardize management of adult inpatients at risk for refeeding syndrome (RFS). To our knowledge, this is the first published study to examine an EMR-integrated order set dedicated to RFS prevention, providing new evidence on its operational impact and potential clinical implications.

### 4.1. Major Findings

Although the implementation of the RROS did not significantly reduce the occurrence or severity of RFS, its integration into the EMR proved highly feasible and was adopted immediately, as evidenced by the absence of a washout period. This rapid uptake underscores the practicality of embedding ASPEN-based guidance directly into ordering workflows. While clinical outcomes remained unchanged, likely because providers were already following core ASPEN recommendations, the RROS offers meaningful operational advantages.

By automating key components of RFS management, the RROS reduces the risk of omission errors, such as failing to recheck labs or initiate timely electrolyte replacement. It also ensures that essential micronutrients, including thiamine and folate, are ordered consistently. These features promote workflow efficiency and standardization, which is particularly valuable for new providers, during periods of high census, or when staffing is limited. In these scenarios, the RROS serves as a safety net, supporting adherence to best practices without relying solely on individual clinician memory or experience.

Despite no significant differences in RFS occurrence, some secondary outcomes did show significant results. First, the post-RROS group demonstrated significantly lower initial EN rates, making it more consistent with the RROS required 10 mL/h starting rate. Second, electrolyte boluses were more common on EN Day 0 in the post-RROS group. This likely reflects earlier identification and correction of deficiencies, as the order set placed electrolyte monitoring and replacement orders simultaneously with EN initiation. In contrast, Day 2 K and Mg labs were more frequently drawn in the pre-RROS group, likely reflecting either clinician-driven variability before standardized monitoring or differing clinical needs between study groups. Together, these results suggest that while the RROS may not alter biochemical outcomes in settings where guidelines are already followed, it enhances reliability, reduces variability, and supports safer EN initiation.

### 4.2. Diagnostic Challenges in RFS

Interpreting RFS occurrence requires caution because diagnostic ambiguity remains a major challenge in the field. Reported incidence varies dramatically, 12.9% to 65.9% in older adults depending on the diagnostic criteria applied, 17.1% in neurocritically ill patients, and even higher in certain malnourished populations [4,5]. The 2020 ASPEN criteria aimed to reduce diagnostic variability, but its reliance on laboratory thresholds alone misses clinical signs and depends heavily on timing of repeat labs, without accounting for other factors affecting electrolytes. These guidelines require clinical judgement, which could not be assessed in this retrospective study.

The high RFS rate (>90%) observed in both groups reflects the use of ASPEN criteria, which define RFS as any ≥10% reduction in laboratory Phos, K, or Mag levels within five days of EN initiation. While this approach promotes early detection, it also captures minor, clinically insignificant electrolyte changes that may not lead to adverse outcomes. In our cohort, a substantial proportion of cases were mild, whereas moderate and severe reductions were less frequent, underscoring the variability in clinical significance.

Applying ASPEN criteria improved standardization but introduced potential misclassification. Prior evaluations show these criteria often classify large numbers of enterally fed patients as having mild RFS, though the clinical relevance of this designation remains uncertain [7]. Because retrospective data capture only once-daily labs and omit clinical context, RFS may have been underdiagnosed if electrolyte drops occurred between draws or were corrected before repeat measurements. Conversely, small, transient reductions may have been labeled as RFS, contributing to overdiagnosis. These limitations, also noted in previous literature, complicate interpretation and likely explain the difficulty in detecting meaningful between-group differences [4,5].

### 4.3. Strengths and Limitations

This study has several notable strengths. It represents the largest evaluation to date of an EMR-embedded RROS and includes a medically complex population from a large, academic medical center, which increases its applicability. The use of diagnostic recommendations provided by ASPEN helped to ensure a standardized approach to identifying RFS across more than 480 patients. Additionally, a detailed assessment of feeding rates, electrolyte trends, and replacement practices over the first five days of EN provides an important operational perspective that has been largely absent from the RFS literature.

However, there are many limitations that warrant further consideration. As a quality improvement project without randomization, results cannot be interpreted as causal. Although groups were generally comparable in age, length of stay, and malnutrition screening scores, notable differences existed in BMI, malnutrition prevalence, and ICU admission rates. These differences reflect the study design and real-world application of the RROS but may have introduced confounding that could influence interpretation of the intervention’s effect. Importantly, this variability provides insight into clinical practice: providers applied the RROS even in patients without a malnutrition diagnosis, indicating potential for expanded use beyond traditional high-risk populations. Data extraction allowed only one laboratory value and one EN order per day, preventing assessment of intra-day fluctuations and potentially obscuring electrolyte shifts indicative of RFS. Inclusion of patients on continuous renal replacement therapy (CRRT) or hemodialysis (whose electrolytes are profoundly influenced by renal replacement therapy), or other clinical conditions impacting electrolyte levels may have diluted detectable differences related to RFS and secondary outcomes. Finally, because a Clinical Nutrition team member was involved in developing the order set, some implementation bias is possible, although such real-world overlap between protocol creation and clinical practice is typical of quality-improvement projects.

### 4.4. Implications and Future Directions

This study demonstrates that embedding RFS protocols into EMR workflows is practical, immediately utilized, and likely improves safety and efficiency. Hospitals with variable RD coverage, high provider turnover, or limited familiarity with ASPEN guidelines may benefit most. Future research should explore whether EMR-based tools combined with predictive analytics or machine learning can further enhance early risk identification and personalized management strategies [8,9].

## 5. Conclusions

Although the EMR-embedded Refeeding Risk Order Set (RROS) did not significantly reduce the occurrence or severity of refeeding syndrome (RFS), this study shows that integrating ASPEN-based guidance into the EMR is feasible and was adopted immediately. Automating key elements such as electrolyte monitoring, micronutrient supplementation, and conservative feeding initiation reduces the risk of errors and promotes consistent care. These benefits improve workflow efficiency and support providers during high census periods, limited staffing, or when experience varies. While clinical outcomes may not change where best practices are already followed, embedding protocols into EMR workflows enhances safety and reliability. Future research should explore combining EMR tools with predictive analytics to optimize early risk identification and individualized management.

## Figures and Tables

**Table 1:** Baseline characteristics of study population.

	Pre-Protocol	Post-Protocol	*p*-Value
Age	61.8 ± 13.7	59.8 ± 15.9	0.14
BMI	23.9 ± 6.9	27.5 ± 19.5	0.017 *
Malnutrition Status			<0.001 *
No Malnutrition	19 (9.2)	104 (37.8)	
Mild	27 (13.0)	19 (6.9)	
Moderate	47 (22.7)	46 (16.7)	
Severe	114 (55.1)	106 (38.6)	
MST Score			0.49
0	47 (33.8)	62 (35.8)	
1	14 (10.1)	28 (16.2)	
2	18 (13.0)	18 (10.4)	
3	17 (12.2)	26 (15.0)	
4	12 (8.6)	13 (7.5)	
5	18 (13.0)	15 (8.7)	
6	13 (9.4)	11 (6.4)	
LOS	22.3 ± 29.1	24.0 ± 27.0	0.5
ICU Admission			0.02 *
No	53 (25.6)	47 (17.1)	
Yes	154 (74.4)	228 (82.9)	

Note: Analysis of baseline characteristics between study groups. Student *t*-tests were utilized when comparing means and are presented as mean and standard deviation. Chi-squared tests were utilized when comparing frequency and are presented as frequency (%). * Indicates significant *p*-value < 0.05.

**Table 2:** RFS frequency and severity between groups.

	Pre-Protocol	Post-Protocol	*p*-Value
RFS Occurrence	191 (92.3)	251 (91.3)	0.694
RFS Severity			0.535
No RFS	16 (7.7)	24 (8.7)	
Mild	42 (20.3)	62 (22.6)	
Moderate	50 (24.2)	76 (27.6)	
Severe	99 (47.8)	113 (41.1)	

Note: Chi-squared test used to compare frequency of RFS between groups. Additional analysis was run to compare the severity of RFS between groups. Data presented as frequency (%).

**Table 3:** Average change in electrolyte levels each day between groups.

	Pre-Protocol	Post-Protocol	*p*-Value
Avg. Δ Electrolyte Level Day 0–1			
K	−0.006 ± 0.54	−0.049 ± 0.48	0.353
Mg	0.037 ± 0.33	0.032 ± 0.33	0.861
Phos	−0.311 ± 0.94	−0.157 ± 1.27	0.146
Avg. Δ Electrolyte Level Day 1–2			
K	−0.030 ± 0.51	−0.038 ± 0.46	0.869
Mg	−0.028 ± 0.34	−0.020 ± 0.30	0.794
Phos	−0.137 ± 0.80	−0.268 ± 0.93	0.125
Avg. Δ Electrolyte Level Day 2–3			
K	0.051 ± 0.47	0.076 ± 0.41	0.567
Mg	−0.026 ± 0.30	−0.037 ± 0.25	0.685
Phos	0.046 ± 0.88	−0.116 ± 0.80	0.060
Avg. Δ Electrolyte Level Day 3–4			
K	0.086 ± 0.37	0.043 ± 0.43	0.303
Mg	0.030 ± 0.29	−0.018 ± 0.24	0.079
Phos	0.166 ± 0.77	0.254 ± 0.93	0.338
Avg. Δ Electrolyte Level Day 4–5			
K	0.062 ± 0.36	−0.011 ± 0.43	0.093
Mg	−0.001 ± 0.29	0.004 ± 0.28	0.858
Phos	0.033 ± 0.72	0.083 ± 0.90	0.595

Note: Average change in electrolyte levels each day compared between groups using a student *t*-test. Results presented as average and standard deviation.

**Table 4:** Number of lab draws each day between groups.

EN Day	Pre-Protocol	Post-Protocol	*p*-Value
Day 0			
K	207 (100)	275 (100)	-
Mg	207 (100)	275 (100)	-
Phos	207 (100)	275 (100)	-
Day 1			
K	205 (99.0)	274 (99.6)	0.580 ^
Mg	206 (99.5)	271 (98.6)	0.397 ^
Phos	204 (98.6)	271 (98.6)	1.000 ^
Day 2			
K	202 (97.6)	256 (93.09)	0.033 ^*
Mg	197 (95.2)	245 (89.09)	0.017 *
Phos	190 (91.8)	244 (88.73)	0.267
Day 3			
K	182 (87.9)	250 (90.9)	0.831
Mg	177 (85.5)	233 (84.7)	0.812
Phos	175 (84.5)	229 (83.3)	0.708
Day 4			
K	166 (80.2)	224 (81.5)	0.727
Mg	159 (76.8)	215 (78.2)	0.721
Phos	157 (75.9)	209 (76.0)	0.969
Day 5			
K	157 (75.9)	207 (75.3)	0.885
Mg	151 (73.0)	194 (70.6)	0.563
Phos	147 (71.0)	184 (66.9)	0.336

Comparison of the number of lab draws for K, Mg, and Phos on each day between groups. Data presented as frequency (%). * Indicates significant *p*-value < 0.05. ^ Indicates use of Fisher’s Exact Test when at least 1 group had a value of less than 5. EN: Enteral Nutrition; K: Potassium; Mg: Magnesium; Phos: Phosphorus.

**Table 5:** Number of electrolyte boluses administered between groups.

Pre-Protocol						Post-Protocol					*p*-Value
# of E. Bolus	0 E. Bolus	1 E. Bolus	2 E. Bolus	3 E. Bolus	4 E. Bolus	0 E. Bolus	1 E. Bolus	2 E. Bolus	3 E. Bolus	4 E. Bolus	
Day 0	143	52	9	0	3	176	79	13	7	0	0.027 *^
Day 1	155	47	4	1	0	203	65	6	1	0	0.979 ^
Day 2	160	42	3	2	0	210	53	11	1	0	0.332 ^
Day 3	152	49	4	1	1	209	59	3	4	0	0.539 ^
Day 4	171	31	4	1	0	210	55	7	1	2	0.406 ^
Day 5	175	29	3	0	0	213	50	10	1	1	0.181 ^

Frequency of electrolyte boluses (E. Bolus) administered each day were compared categorically using chi squared tests. * Indicates significant *p*-value < 0.05. ^ Indicates use of Fisher’s Exact Test when at least 1 group had a value of less than 5. E = electrolyte bolus.

**Table 6:** Average EN start rate and advancement rate compared between groups.

	Pre-Protocol	Post-Protocol	*p*-Value
EN Start Rate (mL/h)	18.3 ± 14.0	15.7 ± 7.9	0.045 *
EN Adv 1	14.9 ± 11.8	14.7 ± 9.3	0.834
EN Adv 2	17.9 ± 14.3	18.0 ± 12.2	0.923
EN Adv 3	7.4 ± 18.5	5.4 ± 17.3	0.536
EN Adv 4	10.8 ± 24.3	7.4 ± 21.8	0.499
EN Adv 5	6.5 ± 14.1	11.4 ± 20.9	0.234

Note: Average EN starting and advancement rate each day compared utilizing student *t*-tests. Presented as mean (average) and standard deviation. * Indicates significant *p*-value < 0.05. EN: Enteral Nutrition; mL/h: milliliters per hour; Adv: Advance.

## Data Availability

The data underlying this study are not publicly available due to institutional privacy policies and the presence of protected health information within the electronic medical record. As this project was conducted as a quality improvement initiative, the de-identified dataset was generated internally for clinical operations and cannot be shared externally. No publicly archived datasets were created or analyzed in this study.

## Abbreviations

The following abbreviations are used in this manuscript:

RFS	Refeeding syndrome
ASPEN	American Society for Parenteral and Enteral Nutrition
EN	Enteral nutrition
EMR	Electronic medical record
RROS	Refeeding Risk Order Set
RD	Registered dietitian
mg	Milligram
g	Gram
BMP	Basic metabolic panel
Mg	Magnesium
Phos	Inorganic Phosphate
dL	Deciliter
mL	Mililiter
h	Hour
BMI	Body mass index
MST	Malnutrition Screening Tool
LOS	Length of hospital stay
ICU	Intensive Care Unit
ANOVA	Analysis of variance
CRRT	Continuous Renal Replacement Therapy
ML	Machine learning
